# Upregulated miR-146b-3p predicted rheumatoid arthritis development and regulated TNF-α-induced excessive proliferation, motility, and inflammation in MH7A cells

**DOI:** 10.1186/s12865-024-00629-9

**Published:** 2024-06-20

**Authors:** Linxiao Ma, Huijie Liu, Ping Shao, Qian Lv

**Affiliations:** Department of Rheumatology, The First People’s Hospital of Lianyungang, No.6 Zhenhua East Road, Lianyungang, 222000 Jiangsu China

**Keywords:** Autoimmune disease, Biomarker, Diagnosis, Development, Fibroblast-like synoviocytes, miRNA, Target genes

## Abstract

**Background:**

Rheumatoid arthritis (RA) is a chronic immune system disease with a high disability rate threatening the living quality of patients. Identifying potential biomarkers for RA is of necessity to improve the prevention and management of RA.

**Objectives:**

This study focused on miR-146b-3p evaluating its clinical significance and revealing the underlying regulatory mechanisms.

**Materials and methods:**

A total of 107 RA patients were enrolled, and both serum and synovial tissues were collected. Another 78 osteoarthritis patients (OA, providing synovial tissues), and 72 healthy individuals (providing serum samples) were enrolled as the control group. The expression of miR-146b-3p was analyzed by PCR and analyzed with ROC and Pearson correlation analyses evaluating its significance in diagnosis and development prediction of RA patients. In vitro, MH7A cells were treated with TNF-α. The regulation of cell proliferation, motility, and inflammation by miR-146b-3p was assessed by CCK8, Transwell, and ELISA assays.

**Results:**

Significant upregulation of miR-146b-3p was observed in serum and synovial tissues of RA patients, which distinguished RA patients and were positively correlated with the erythrocyte sedimentation rate (ESR), C-reactive protein (CRP), anti-cyclic citrullinated peptide antibodies (anti-CCP), and rheumatoid factor (RF) of RA patients. TNF-α promoted the proliferation and motility of MH7A cells and induced significant inflammation in cells. Silencing miR-146b-3p alleviated the effect of TNF-α and negatively regulated the expression of *HMGCR*. The knockdown of *HMGCR* reversed the protective effect of miR-146b-3p silencing on TNF-α-stimulated MH7A cells.

**Conclusions:**

Increased miR-146b-3p served as a biomarker for the diagnosis and severity of RA. Silencing miR-146b-3p could suppress TNF-α-induced excessive proliferation, motility, and inflammation via regulating *HMGCR* in MH7A cells.

**Supplementary Information:**

The online version contains supplementary material available at 10.1186/s12865-024-00629-9.

## Background

Rheumatoid arthritis (RA) is a well-known and common autoimmune disease with synovitis as the major characteristic. The clinical symptoms of RA mainly behaved as symmetrical and progressive chronic multiarticular inflammation, especially in small joints, such as hands and wrists, were evident. Angiogenesis induced by the extensive proliferation of synovial cells and irreversible joint injury are the typical pathological features of RA. The incidence of RA is global, and the new cases and disability rates are increasing gradually in China. The pathological mechanism of RA remains unclear [1]. The diagnosis of RA is primarily through biochemical indicators, such as the increasing erythrocyte sedimentation rate, increasing C-reactive protein, and positive anti-cyclic citrullinated peptide antibodies, confirmed by imaging examinations [2, 3]. Rheumatoid factor (RF) and anti-cyclic citrullinated peptide antibodies (anti-CCP) are commonly used antibodies for the diagnosis of RA, which is associated with RA progression and indicates the increasing potential of lifelong medication [4, 5]. Fibroblast-like synoviocytes (FLSs), existing in the synovium of the joint, are the critical component of synovial tissues and possess similar characteristics to fibroblasts [6]. The extensive proliferation of FLSs is the initial link of RA and continuously deteriorates disease progression [7]. Inflammation could induce the activation of FLSs, which upregulates the levels of vascular adhesion molecules, binding with the ligands in the chondrocyte or extracellular matrix, mediating the connections between cells and extracellular matrix or other cells, and therefore regulate cell biological behaviors [8]. In addition, activated FLSs are involved in the various pathological changes during RA, such as synovium-invaded cartilage tissues, secreted inflammatory cytokines, and matrix metal albumin-destroyed bone tissues [9]. Therefore, an increasing number of studies have also focused on FLSs aiming to explore novel therapeutic strategies to reduce the side effects of anti-rheumatoid drugs. Exploring regulators for FLSs is of great significance.

Non-coding RNAs (ncRNAs) account for over 90% of the human genome, which promotes the development of a new field in epigenetics [10, 11]. microRNAs (miRNAs) are a critical member of the ncRNA family, playing vital roles in cell cycle, apoptosis, differentiation, and immune response. miRNAs have also been reported to mediate the course of disease in RA. The regulatory mechanisms underlying the function of miRNAs have been illustrated including regulating the physiological function of Treg cells, regulating B cells, and regulating cytokines [12, 13]. Moreover, functional miRNAs were also suggested as therapeutic targets of non-steroidal anti-inflammatory drugs, anti-rheumatic drugs, immunosuppressants, and herbs. A recent study explored several candidate miRNAs for the diagnosis of RA, psoriatic arthritis, and ankylosing spondylitis, and miR-146b-3p showed great significance [14]. The significance of miR-146b-3p has been confirmed in human cancers and inflammation regulation [15–18]. miR-146b-3p was hypothesized to regulate inflammation and therefore mediate the progression of RA.

In mechanism, miRNAs could bind with the 3’UTR of target genes and further regulate their expression and function. From public databases, 3-hydroxy-3-methylglutaryl-CoA reductase (HMGCR) was predicted as the downstream target of miR-146b-3p, which was previously demonstrated to be associated with the therapy of autoimmune diseases [19, 20]. Hence, whether HMGCR mediates the function of miR-146b-3p in RA was also evaluated in this study, aiming to complete its regulatory mechanism.

This study investigated the clinical significance of miR-146b-3p in RA occurrence and development and revealed the molecular mechanism underlying the regulatory effect of miR-146b-3p, aiming to provide reference to elucidate the pathogenesis of RA and optimize the therapy strategies.

## Methods

### Patient inclusion and sample collection

The study enrolled 107 RA patients, 78 osteoarthritis (OA) patients, and 72 healthy individuals from The First People’s Hospital of Lianyungang during 2019–2022. OA patients and healthy individuals were set as control groups providing synovial tissues and serum samples, respectively. This study had been approved by the Ethics Committee of The First People’s Hospital of Lianyungang, and all participants signed informed consent. The inclusion and exclusion criteria for each group were as follows.

RA and OA patients were diagnosed according to the American College of Rheumatology Guideline for rheumatoid arthritis and osteoarthritis, respectively. Patients with complications of other autoimmune diseases, cardiovascular and cerebrovascular diseases, endocrine diseases, and infectious diseases were excluded. Both RA and OA patients received synovectomy, and the synovial tissues were collected during the surgery. Collected tissues were frozen with liquid nitrogen and stored at -80 °C until following analyses.

Healthy individuals were enrolled in the physical examination center of our hospital in the same period. Healthy individuals were excluded for any diseases and with the matched age and gender composition of RA and OA patients.

Fasting elbow venous blood samples were collected from healthy individuals and RA patients the next morning of their enrollment. Collected blood samples were stood at room temperature for 30 min followed by centrifugation at 10,000 g for 15 min to isolate serum.

### Cell culture

Human FLSs, MH7A cells, were purchased from ATCC and maintained with RPMI 1640 culture medium (Hyclone, USA) supplemented with 10% FBS (Gibco, USA) and penicillin/streptomycin (v: v 1:100, Sigma, USA). Cell culture was conducted at 37 °C with a humidity of 95% and 5% CO_2_.

### Cell transfection and treatment

Cells were transfected with miR-146b-3p inhibitor or negative controls (NC) and small interference RNA of HMGCR (si-HMGCR) using Lipofectamine 3000 (Invitrogen, USA). Cell transfection was performed at room temperature, and transfected cells were available for the following experiments after incubation at 37 °C for 48 h. Transfected cells were treated with TNF-α (10 ng/mL) for 24 h to stimulate inflammation according to previous studies [21, 22]. Cells without any treatments were set as the control group.

### Total RNA extraction

Tissues were washed with pre-cooled PBS solution and then lysed with Trizol reagent (Invitrogen, USA). Cells were digested with 0.25% pancreatic enzymes followed by lysing with Trizol reagent. The mixture was stood on ice for 10 min followed by centrifugation at 13,400 g for 10 min. Then, the upper transparent layer was transferred to another tube mixed with chloroform and stood on ice for 5 min. After centrifugation at 13,400 g for 10 min, the supernatant was further mixed with a mixture of phenol and chloroform (v: v 1:1) and stood at room temperature for 5 min followed by centrifugation at 13,400 g for 10 min. Then, 500 µL isopropanol was added and mixed well. The mixture was finally centrifugated at 13,400 g for 10 min, and the precipitate was total RNA. The purity and concentration of isolated RNA were evaluated by the ratio of OD260/280, which ranged from 1.8 to 2.0 indicating high-quality RNA.

### Real-time quantitative PCR

Total RNA was reverse transcribed to cDNA with the employment of TaqMan MicroRNA Reverse Transcription kit (Applied Biosystem, USA) for miR-146b-3p and High-Capacity cDNA Reverse Transcription kit (Applied Biosystem, USA) for HMGCR according to the manufacturer’s protocol. Amplification was performed on the ABI PCR system, and the relative expression levels were calculated by the 2^−ΔΔCT^ method relative to cel-miR-39 and GAPDH for miR-146b-3p and HMGCR, respectively. The primers have been summarized in Table S1.

### Dual-luciferase reporter assay

The binding sites between miR-146b-3p and HMGCR were predicted from the online databases. Wild-type and mutant-type plasmids were established with the pGL3 plasmid and co-transfected with miR-146b-3p mimic, inhibitor, or negative controls using Lipofectamine 3000 (Invitrogen, USA). The luciferase activity of HMGCR was detected on the Dual-luciferase reporter system (Promega, USA) relative to Renilla.

### MTT assay

Cells were seeded onto 96-well plates and incubated at 37 °C for 24, 48, 72, and 96 h followed by the addition of MTT solution. Cells were incubated for another 4 h after adding MTT solution, and then mixed with DMSO solution. The plates were incubated in the dark for 20 min, and the absorbance at 490 nm was measured with a microplate reader (Molecular Devices, USA).

### Transwell assay

Cells were seeded onto the upper chamber of the 24-well transwell plates supplied with an FBS-free culture medium. The lower chamber was filled with a completed culture medium containing 10% FBS. The plates were incubated at 37 °C for 48 h, and then cells remaining in the upper chamber were removed. Cells on the subsurface of the transwell chamber were fixed and stained. The number of migrated and invasive cells was counted with an optical microscope (Carl Zeiss, USA).

### Enzyme-linked immunosorbent assay

Cells were seeded onto 96-well plates and treated with corresponding transfection and TNF-α inducement. The supernatant was measured with commercial ELISA kits (Biosource International, USA) according to the manufacturer’s instructions to detect the protein levels of IL-6, IL-8, and IL-1β.

### Statistical analyses

The diagnostic value of miR-146b-3p in RA was evaluated by ROC (AUC > 0.5), and its association with RA patients’ disease conditions was assessed by Pearson correlation analysis (*P* < 0.05). Difference comparison was performed with a student’s t-test (for two groups) and one-way ANOVA (for multiple groups, *P* < 0.05). All statistical analyses were conducted using SPSS 26.0 software and GraphPad Prism 9.0 software.

## Results

### Baseline information of study subjects

The three groups of study subjects possessed matched age and gender composition. Healthy individuals included 27 males and 45 females with an average age of 51.19 ± 7.05 years, while OA patients were composed of 30 males and 48 females with an average age of 52.95 ± 7.07 years. The enrolled RA patients included 41 males and 66 females with an average age of 51.84 ± 7.25 years (*P* > 0.05, Table 1).

**Table 1:** Baseline information of three groups

	Healthy individuals	OA patients	RA patients	*P*-value
Age (years)	51.19 ± 7.05	52.95 ± 7.07	51.84 ± 7.25	0.311
Gender (male/female)	27/45	30/48	41/66	0.991
BMI	25.01 ± 2.17	24.29 ± 1.72	24.51 ± 2.76	0.151
ESR	11.08 ± 2.66	28.64 ± 6.60	46.44 ± 6.62	< 0.001
CRP	0.05 ± 0.03	1.06 ± 0.17	1.58 ± 0.42	< 0.001
Anti-CCP	-	1.91 ± 0.80	37.97 ± 16.57	< 0.001
RF	-	7.74 ± 2.85	63.95 ± 27.14	< 0.001
Disease duration	-	52.47 ± 18.54	58.31 ± 24.60	0.083

BMI: body mass index, kg/m^2^; ESR: erythrocyte sedimentation rate, mm/h; CRP: C-reactive protein, mg/dL; anti-CCP: anti-cyclic citrullinated peptide antibodies, IU/mL; RF: rheumatoid factor, IU/Ml

OA and RA patients showed significantly increasing ESR and CRP compared with healthy individuals. The levels of anti-CCP and RF of RA patients were significantly higher than that of OA patients (*P* < 0.001). The disease duration between OA and RA patients showed no significant difference (*P* > 0.05, Table 1).

### Significance of miR-146b-3p in distinguishing RA patients

**Fig. 1:**
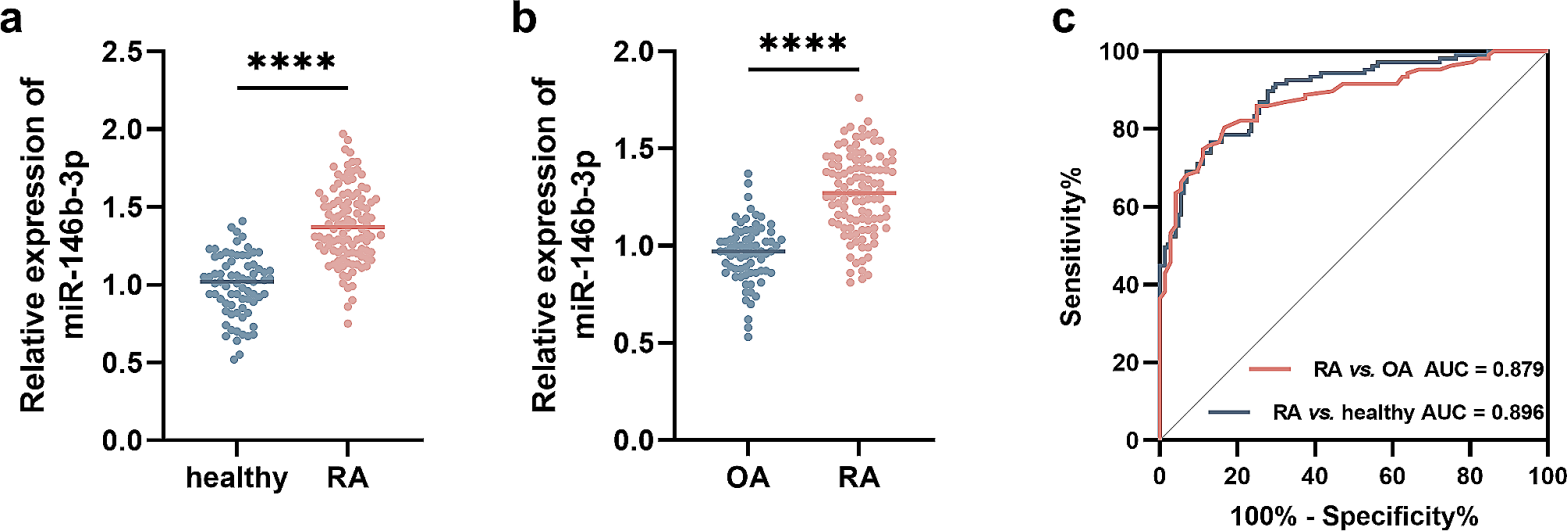
Expression of miR-146b-3p in serum (**a**) and synovial tissues (**b**) of RA and its significance in distinguishing RA patients (**c**). ****P < 0.0001

Compared with healthy individuals, RA patients showed a higher serum miR-146b-3p (Fig. 1a). For its expression in synovial tissues, RA patients showed a higher expression than in OA patients (Fig. 1b). Abnormal expression of miR-146b-3p in the serum and synovial tissues of RA patients showed dramatical significance in discriminating RA patients from healthy individuals (AUC = 0.896, 95% = 0.852–0.941) and OA patients (AUC = 0.879, 95% CI = 0.829–0.928, Fig. 1c).

### Correlation of miR-146b-3p with the clinicopathological features of RA patients

**Fig. 2:**
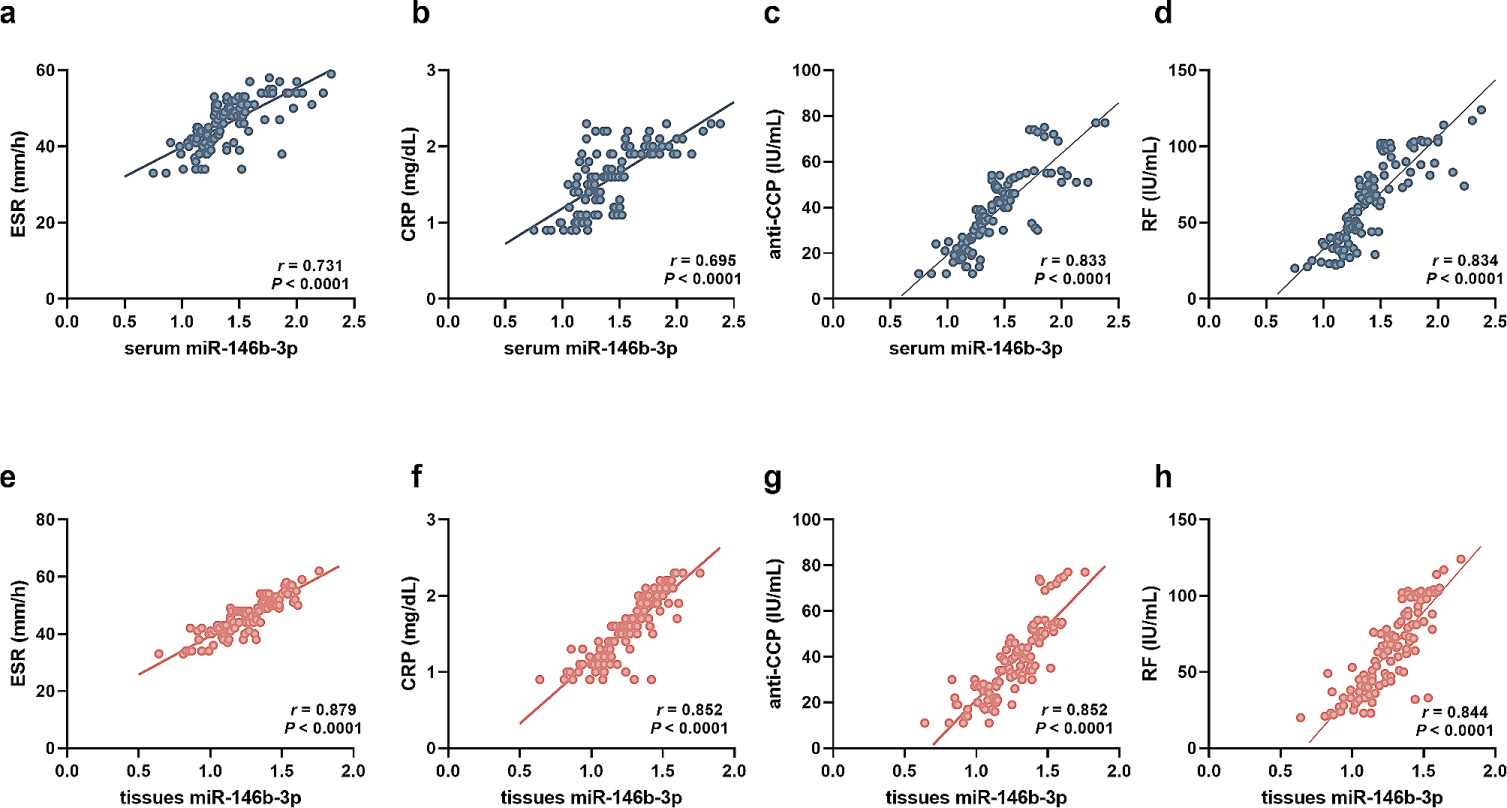
Correlation of serum (**a**-**d**) and synovial tissues (**e**-**h**) miR-146b-3p with the ESR (**a** and **e**), CRP (**b** and **f**), anti-CPP (**c** and **g**), and RF (**d** and **h**) of RA patients

Increasing serum miR-146b-3p levels in RA patients showed significantly positive correlation with the ESR (*r* = 0.731, Fig. 2a), CRP (*r* = 0.695, Fig. 2b), anti-CCP (*r* = 0.833, Fig. 2c), and RF (*r* = 0.834, Fig. 2d) of patients (*P* < 0.0001). Similar correlations were also observed in the tissue miR-146b-3p levels. The higher expression of miR-146b-3p in synovial tissues was also associated with the increasing ESR (*r* = 0.879, Fig. 2e), CRP (*r* = 0.852, Fig. 2f), anti-CCP (*r* = 0.852, Fig. 2g), and RF (*r* = 0.844, Fig. 2h) of RA patients (*P* < 0.0001).

### Regulatory effect of miR-146b-3p on HMGCR in TNF-α-stimulated FLSs

**Fig. 3:**
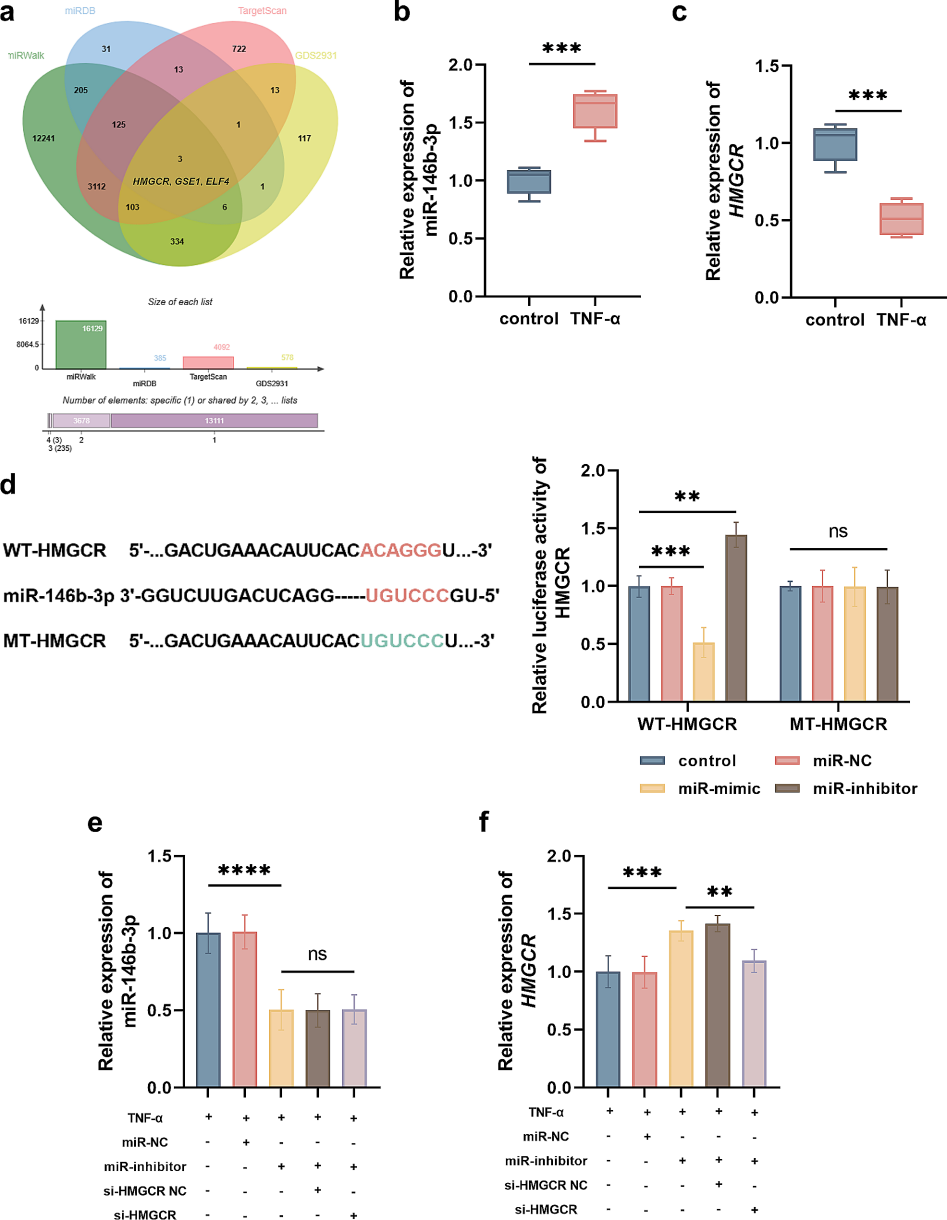
Targeting interaction between miR-146b-3p and HMGCR. **a**. Prediction of miR-146b-3p targets and dysregulated genes from miRWalk, miRDB, TargetScan databases, and GDS2931 dataset. **b**-**c**. Expression of miR-146b-3p (**b**) and HMGCR (**c**) in MH7A cell. **d.** Regulation of miR-146b-3p on the luciferase activity of HMGCR. **e-f**. Expression of miR-146b-3p (**e**) and HMGCR (**f**) in TNF-α-stimulated FLSs. ^ns^*P* > 0.05, ^**^P < 0.01, ^***^P < 0.001, ^****^P < 0.0001

The downstream targets of miR-146b-3p were predicted from the three public databases, miRWalk, miRDB, and TargetScan, and the enriched targets further intersected with the GEO dataset GDS2931, a dataset screen dysregulated mRNAs in synovial tissues of RA patients compared with OA patients. *HMGCR*, *GSE1*, and *ELF4* were finally identified as key targets of miR-146b-3p that are differentially expressed in RA (Fig. 3a). In FLSs treated with TNF-α, HMGCR was suppressed by the overexpression of miR-146b-3p and enhanced by its silencing (Figure S1a), while GSE1 (Figure S1b) and ELF4 (Figure S1c) were not affected.

In TNF-α-stimulated FLSs, significant upregulation of miR-146b-3p (Fig. 3b) and downregulation of HMGCR (Fig. 3c) were observed compared with untreated cells. Based on the predicted binding sites between miR-146b-3p and HMGCR, the WT-HMGCR and MT-HMGCR vectors were established. Overexpressing miR-146b-3p significantly suppressed the luciferase activity of *HMGCR*, while silencing miR-146b-3p showed an opposite effect (Fig. 3d). miR-146b-3p was silenced in TNF-α-stimulated FLSs by the transfection of miR-146b-3p inhibitor (Fig. 3e), which enhanced the expression of HMGCR (Fig. 3f). The knockdown of HMGCR showed no significant effect on the expression of miR-146-3p but could reverse the increasing expression of HMGCR by miR-146b-3p silencing (Fig. 3e and f).

### Involvement of HMGCR in the regulatory effect of miR-146b-3p on TNF-α-stimulated FLSs

**Fig. 4:**
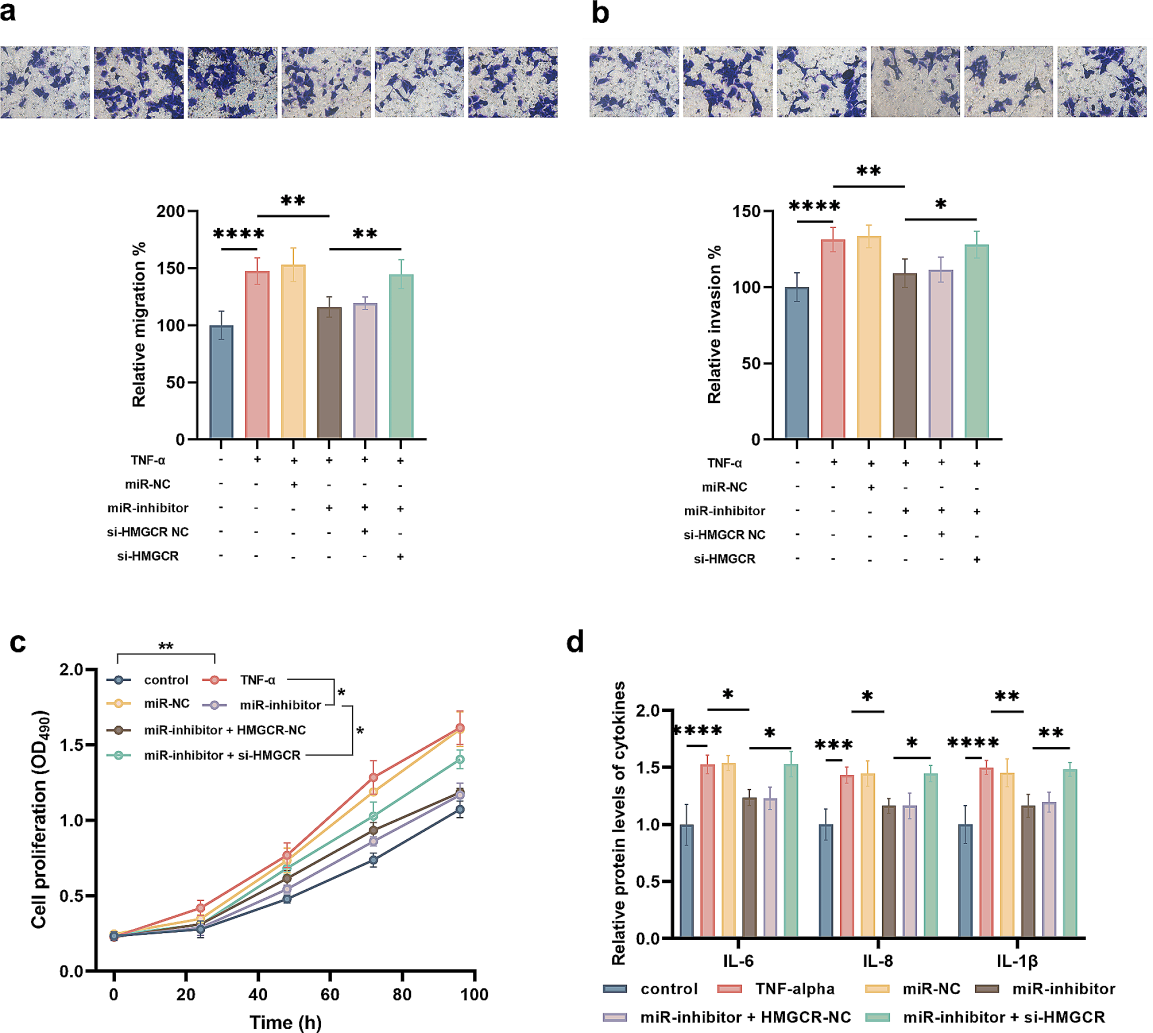
Regulatory effect of the miR-146b-3p/HMGCR axis on the migration (**a**), invasion (**b**), proliferation (**c**), and inflammation (**d**) in TNF-α-stimulated FLSs. ^*^P < 0.05, ^**^P < 0.01, ^***^P < 0.001, ^****^P < 0.0001

Compared with untreated cells, TNF-α treatment led to increased migration (Fig. 4a), invasion (Fig. 4b), and proliferation (Fig. 4c) of FLSs and induced significant inflammatory response (Fig. 4d). Silencing miR-146b-3p suppressed migration, invasion, and proliferation of TNF-α-stimulated FLSs and alleviated inflammations. The knockdown of HMGCR reversed the protective effect of miR-146b-3p silencing on FLSs with the treatment of TNF-α.

## Discussion

The miR-146 family has been identified as a key modulator of immunity and autoimmune diseases. For example, the genetic polymorphisms of miR-146a served as diagnostic biomarkers for systemic lupus erythematosus and regulated disease development via regulating mesenchymal stem cells [23–25]. This study focused on miR-146b-3p, a major member of the miR-146 family that was previously revealed to possess the potential to serve as a biomarker for RA [14]. Blood samples are commonly analyzed for disease diagnosis, while tissue samples are more closely associated with the disease conditions of patients. Herein, the expression of miR-146b-3p was analyzed in both serum and synovial tissues. For the selection of the control group, serum samples were obtained from healthy individuals, but it is hard to obtain synovial tissues from healthy individuals, therefore a group of OA patients were enrolled providing synovial tissues. Significant upregulation of miR-146b-3p was observed in both serum and synovial tissues of RA patients compared with control groups, which distinguish RA patients from healthy individuals and OA patients sensitively and specifically. During the onset of RA, the increase of fibrinogen and globulin would promote ESR, and therefore, ESR has also been demonstrated to possess a close relationship with the severity of RA [26–28]. RF and anti-CCP are widely accepted antibodies for screening RA and are associated with the disease progression of RA. The higher levels of RF and anti-CCP indicate the increasing potential of lifelong medication [4, 5]. Consistently, a significant increase was observed in ESR and CRP of OA and RA patients, and RA patients showed a higher anti-CCP and RF than OA patients. miR-146b-3p expression in serum and synovial tissues showed positive correlations with these severity indicators in RA patients, indicating its positive association with disease severity. Hence, upregulated miR-146b-3p could indicate the occurrence and severe progression of RA.

Abnormal proliferation of FLSs has been considered one of the main pathologies of RA [29]. It was reported that the number of synovial cell layers in RA patients is over three times that of healthy individuals, resulting from the excessive proliferation of FLSs [30, 31]. The proliferation rate of FLSs in RA patients is much faster than that in healthy individuals. The hyperplasia of FLSs was observed before the gathering of inflammatory cells, suggesting that FLSs play a role in the early stage of inflammation. Previously, the inducement on FLSs was performed with LPS, IL-1β, and many other molecules. TNF-α is a major factor inducing inflammation and could activate FLSs, which play critical roles in the development of RA [32]. In this study, FLSs treated with TNF-α showed enhanced proliferation and motility ability, and TNF-α also promoted the secretion of pro-inflammation cytokines. miRNAs have been demonstrated to play vital roles in regulating FLS injury. For example, overexpressing miR-27b in TNF-α-treated MH7A cells significantly facilitated cell apoptosis [33]. miR-496 regulated the proliferation of IL-1β-induced FLSs via modulating NF-kB signaling [34]. Additionally, in TNF-α-stimulated FLSs, significant upregulation of miR-146b-3p was observed, and silencing miR-146b-3p could suppress TNF-α induced proliferation, migration, invasion, and inflammation.

miRNAs could bind with the 3’UTR of genes and therefore regulate their functions [35]. Among target genes of miR-146b-3p, *HMGCR*, *GSE1*, and *ELF4* were identified to be dysregulated in RA from a GEO dataset, and only *HMGCR* was negatively regulated by miR-146b-3p in TNF-α-stimulated FLSs. TNF-α induced significant downregulation of *HMGCR* in FLSs, and the knockdown of *HMGCR* could reverse the protective effect of miR-146b-3p silencing on FLSs, suggesting the involvement of *HMGCR* in the regulatory effect of miR-146b-3p on TNF-α-stimulated FLSs.

However, there was a lack of validation in the serum of OA patients, which should attract special attention in future studies. The clinical findings lack a validation with larger sample size due to the limited scale and number of research centers. To promote the clinical application of the identified novel biomarkers, further clinical confirmation, especially investigations with larger sample sizes from multiple centers, is necessary. Additionally, deep mechanism investigations are needed to complete the regulatory network of miR-146b-3p and provide more direct therapeutic targets for RA.

## Conclusions

In conclusion, upregulated miR-146b-3p in RA screened disease occurrence and development. Silencing miR-146b-3p suppressed TNF-α-induced excessive cell proliferation, motility, and inflammation in FLSs via negatively regulating *HMGCR*.

### Electronic supplementary material

Below is the link to the electronic supplementary material.

Supplementary Material 1

Supplementary Material 2

## Data Availability

The datasets used and/or analysed during the current study are available from the corresponding author on reasonable request.

## Abbreviations

RA: Rheumatoid arthritis
FLSs: Fibroblast-like synoviocytes
ncRNAs: Non-coding RNAs
miRNAs: MicroRNAs
HMGCR: 3-hydroxy-3-methylglutaryl-CoA reductase
OA: Osteoarthritis
NC: Negative controls
si-HMGCR: Small interference RNA of HMGCR
