# Supplementary figures and images for: Upregulated miR-146b-3p predicted rheumatoid arthritis development and regulated TNF-α-induced excessive proliferation, motility, and inflammation in MH7A cells
Source: BMC Immunol. 2024 Jun 20;25:36. doi: 10.1186/s12865-024-00629-9 (PMC11188492; doi:10.1186/s12865-024-00629-9)

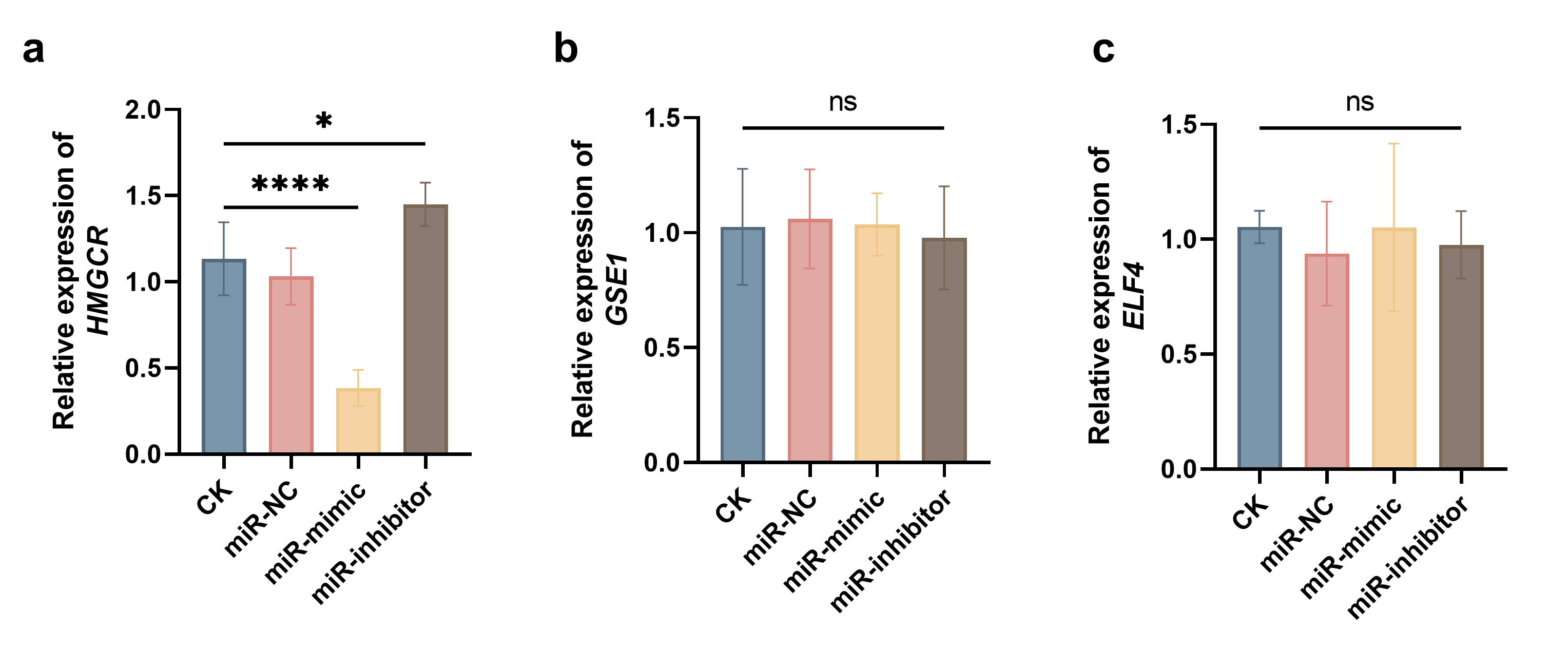

Supplement: Supplementary file 1 — Supplementary Material 1 [file 12865_2024_629_MOESM1_ESM.tif]
