# Supplementary material for: Upregulated miR-146b-3p predicted rheumatoid arthritis development and regulated TNF-α-induced excessive proliferation, motility, and inflammation in MH7A cells
Source: BMC Immunol. 2024 Jun 20;25:36. doi: 10.1186/s12865-024-00629-9 (PMC11188492; doi:10.1186/s12865-024-00629-9)
Supplement: Supplementary file 2 — Supplementary Material 2 [file 12865_2024_629_MOESM2_ESM.docx]

Table S1. Sequences of primers used in PCR

| Primer | Sequences |
| --- | --- |
| miR-146b-3p forward | 5'-GCCCUGUGGACUCAGUUCUG-3' |
| miR-146b-3p reverse | 5'-CTCTACAGCTATATTGCCAGCCAC-3' |
| HMGCR forward | 5'-ATGGAATTCCATGGCTGGGAGCATAGGAG-3' |
| HMGCR reverse | 5'-TCCTTGAACACCTAGCATCTGC-3' |
| Cel-miR-39 (MI0000010) forward | 5'-UCACCGGGUGUAAAUCAGCUUG-3' |
| Cel-miR-39 (MI0000010) reverse | 5'-TCACCGGGTGTAAATCAGCTTG-3' |
| GAPDH (NM_001101) forward | 5'-GGAGTCAACGGATTTGGTCG-3' |
| GAPDH (NM_001101) reverse | 5'-GGAATCATATTGGAACATGTAAACC-3' |
